# Correlation study on microbial communities and volatile flavor compounds in cigar tobacco leaves of diverse origins

**DOI:** 10.1007/s00253-024-13032-6

**Published:** 2024-02-26

**Authors:** Haiqing Wang, Dongfeng Guo, Mingzhu Zhang, Guanglong Wu, Yaqi Shi, Jinglong Zhou, Naihong Ding, Xiangsong Chen, Xingjiang Li

**Affiliations:** 1 Anhui Fermented Food Engineering Research Center, School of Food and Biological Engineering, Hefei University of Technology, Danxia Road 485#, 230601 Hefei City, Anhui Province People’s Republic of China; 2 China Tobacco Anhui Industrial Co., Ltd, Huangshan Road 606#, 230088 Hefe City, Anhui Province People’s Republic of China; 3 Institute of Plasma Physics, Hefei Institutes of Physical Science, Chinese Academy of Sciences, Hefei City, 230009 Anhui Province People’s Republic of China

**Keywords:** Cigar tobacco leaves, Bacteria, Fungi, Volatile flavor compounds, Flavor

## Abstract

**Abstract:**

To elucidate the significant influence of microorganisms on geographically dependent flavor formation by analyzing microbial communities and volatile flavor compounds (VFCs) in cigar tobacco leaves (CTLs) obtained from China, Dominica, and Indonesia. Microbiome analysis revealed that the predominant bacteria in CTLs were *Staphylococcus*, *Aerococcus*, *Pseudomonas*, and *Lactobacillus*, while the predominant fungi were *Aspergillus*, *Wallemia,* and *Sampaiozyma*. The microbial communities of CTLs from different origins differed to some extent, and the diversity and abundance of bacteria were greater than fungi. Metabolomic analysis revealed that 64 VFCs were identified, mainly ketones, of which 23 VFCs could be utilized to identify the geographical origins of CTLs. Sixteen VFCs with OAV greater than 1, including cedrol, phenylacetaldehyde, damascone, beta-damascone, and beta-ionone, play important roles in shaping the flavor profile of CTLs from different origins. Combined with the correlation analysis, bacterial microorganisms were more closely related to key VFCs and favored a positive correlation. *Bacillus*, *Vibrio*, and *Sphingomonas* were the main flavor-related bacteria. The study demonstrated that the predominant microorganisms were essential for the formation of key flavor qualities in CTLs, which provided a theoretical reference for flavor control of CTLs by microbial technology.

**Key points:**

• *It is the high OAV VFCs that determine the flavor profile of CTLs.*

• *The methylerythritol phosphate (MEP) pathway and the carotenoid synthesis pathway are key metabolic pathways for the formation of VFCs in CTLs.*

• *Microbial interactions influence tobacco flavor, with bacterial microorganisms contributing more to the flavor formation of CTLs.*

**Graphical Abstract:**

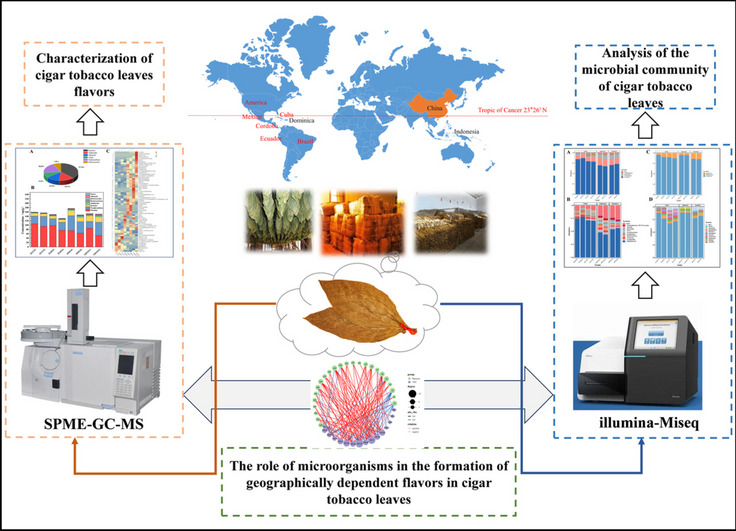

**Supplementary Information:**

The online version contains supplementary material available at 10.1007/s00253-024-13032-6.

## Introduction

Cigars are tobacco products rolled from fermented cigar tobacco leaves (CTLs), which are sought after by tobacco enthusiasts for their potent aroma and distinctive flavor (Viola et al. 2016). The flavor profile of cigars is influenced by several factors, including the variety, origin, and production process of CTLs (Morris and Fiala 2015). Specifically, the environment of origins and the fermentation process is very essential for shaping the flavor quality of CTLs (Fan et al. 2023). Cuba, Dominica, Indonesia, Honduras, etc. are areas that produce high-quality CTLs (Zhang et al. 2021b). Sub-tropical and tropical climatic conditions and favorable soil resources create unique conditions for the growth of CTLs and create a wide range of stylistic characteristics. Cuban CTLs are strong, mellow, spicy, and aromatic, Dominican CTLs are highly prized by consumers around the world for their distinctive mellow, smoky, grassy, and slightly sour flavors. Honduran CTLs are known for their strong, spicy, and aromatic flavor (Zheng et al. 2022). In addition to geographical factors, fermentation is an integral part of CTL processing, exerting a substantial influence on the formation of the flavor profile of CTLs. As the main biological driving force in the fermentation process, microorganisms can participate in the decomposition and transformation of macromolecules and aroma precursors in CTLs and contribute to the production of complex aroma substances (Vermote et al. 2023), which have an important impact on the flavor of tobacco. Because fermentation technology and origin factors vary, different CTLs have different functional microorganisms, which also result in different flavors and quality of fermented CTLs (Yan et al. 2021). The investigation of microbial community structure in CTLs and its association with volatile flavor compounds (VFCs) can point out the direction of improving the quality of CTLs.

Previous research on microbial ecology has relied on culture analysis, the study of microorganisms using isolation and identification. This approach covers only those microorganisms that are easy to culture and does not provide a clear picture of the entire microbial communities (Sharma et al. 2023). With the rise of high-throughput technologies, the development of metabolomics provides practical solutions for studying species, genes, proteins, and metabolites in natural ecosystems (El-Liethy et al. 2023; Gallagher et al. 2022). So far, researchers have used the technique extensively for traditional food fermentation microbial community analysis, such as soybean fermentation (Lin et al. 2021), Baijiu brewing (Wu et al. 2023c), and meat storage (Zhang et al. 2023a). High-throughput sequencing technology also facilitates the study of microorganisms in CTLs. Zhang et al. (2021a) analyzed the bacterial community diversity of Hainan H382 CTLs in different fermentation cycles and found that *Staphylococcus* was always the dominant strain in fermentation. Zhang et al. (2023b) studied the changes in microorganisms and enzymes of CTLs during drying and fermentation and found that the dominant microorganisms differed at different stages of the drying and fermentation processes and that the changes in metabolic enzymes occurred mainly at the drying stage. Zhang et al (2024) studied the improvement of tobacco quality and microbial community succession during microbial co-fermentation, showing that the abundant microorganisms in tobacco can improve its quality, and identified *Bacillus* as an important biomarker of the co-fermentation process. By analyzing the bacterial community composition of wrapper, jacket, and core in different origins, it was found that the bacterial diversity of CTLs of different origins for different uses was rich and the structural composition was similar (Yan et al. 2022). These results suggest that the microbial community composition of CTLs may vary considerably across origins and fermentation processes and microorganisms play a large role in shaping the quality of CTLs.

What is more, the combination of microbiome and metabolomics techniques can better analyze the formation mechanism of flavor quality in CTLs. The headspace solid-phase microextraction method can realize the automation of the aroma enrichment process, and have the advantages of convenience and speed (Kaikiti et al. 2023). Headspace solid-phase microextraction–gas chromatography–mass spectrometry (HS–SPME–GC–MS) is widely used for the determination of VFCs in CTLs. Based on HS–SPME–GC–MS, Wu et al. (2023a) investigated the changes of VFCs during stacking and fermentation of CTLs and concluded that the types of VFCs increased through stacking and fermentation and that Maillard reaction products, carotenoid degradation products, and nicotine degradation products were the key VFCs for the enhancement of the quality of CTLs. During fermentation, the dominant microorganisms affected the production and degradation of the main VFCs through enzyme metabolism with the help of the Maillard reaction (Wu et al. 2023b).

Current research on CTLs has focused more on changes in microbial communities and VFCs during the fermentation of CTLs. Although studies on CTLs of different origins have been reported (Zheng et al. 2022), more attention has been paid to the differences in microbial community composition and flavor of CTLs of different origins, lacking excavation of their advantageous microorganisms and key VFCs based on the differences in origins, and the relationship between key microorganisms and VFCs needs to be further studied. Metabolic pathways for the formation of key VFCs in CTLs from different origins also need to be explored. To further elucidate the mechanism of tobacco flavor formation, we investigated CTLs of various origins to explore (i) the microbial community characteristics and flavor compositions, and (ii) the function of microbial populations in flavor creation. Thus, 8 samples of CTL fermentations from 3 different production areas (China, Indonesia, Dominica) were collected. To analyze the microbial community structure and VFC differences of CTLs from different origins by high-throughput sequencing and metabolomic technologies, to explore the key microorganisms and VFCs in CTLs, and to clarify the metabolic pathways of the key VFCs involved in microorganisms. A multivariate statistical approach to reveal microbial interactions and to analyze the relationship between microbial communities and VFCs in CTLs. The results of the study help to identify the key microorganisms that play an important role in the flavor quality of tobacco and provide a theoretical basis for screening and using functional microorganisms to enhance the flavor quality of CTLs.

## Materials and methods

### Sample collection

Eight samples of CTLs were collected from the Mengcheng Cigar Production Department of China Tobacco Anhui Industrial Co., Ltd, including 2 samples from Dominica, 2 samples from Indonesia, and 4 samples from China (Table 1). The CTLs were randomly sampled from the four corners and center of each storage package. Each sample was about 1000 g, then mixed evenly and put into sterile bags for sealing. At the same time, the sample information, sampling time, and sampling place were marked and then stored at − 20 °C until detection.

**Table 1:** Sample information

Place	Sample	Production time	Collection time	Production use	Climate type	Latitude
China	ZGYX1	2022	2022	Filler tobacco	Subtropical monsoon climate	21° 8′ N
	ZGYX2	2022	2022	Filler tobacco		21° 8′ N
	ZGDX3	2022	2022	Filler tobacco		31° 22′ N
	ZGDX4	2022	2022	Filler tobacco		31° 22′ N
Indonesia	YDNXY1	2020	2022	Filler tobacco	Tropical rainforest climate	6° 19′ S
	YDNXY2	2020	2022	Filler tobacco		6° 19′ S
Dominica	DMNJ1	2021	2022	Filler tobacco	Tropical rainforest climate	18° 48′ N
	DMNJ2	2021	2022	Filler tobacco		18° 48′ N

### Microbial community analysis

DNA extraction and amplification: A 250 mL flask containing 5 g of CTL samples and 50 mL of a pH 7.4 PBS solution was oscillated at 4 °C, 2500 rpm for 4 h. The CTL samples were rinsed repeatedly with PBS solution, the total washing solution was collected, and the bacteria solution was enriched with 0.22 μm filter membrane. Obeying the guidelines provided by the manufacturer, the MagPure Soil DNA LQ Kit (Magan) was applied to extract the whole genome. DNA was not amplified before gel electrophoresis. DNA content and integrity were evaluated by agarose gel electrophoresis using the NanoDrop2000. DNA that had been extracted was kept at − 20 °C until it was processed. Three biological replicates were performed for each sample. Using barcoded primers and Takara Ex Taq (Takara), the extracted DNA was utilized as a template for PCR amplification of the bacterial 16S rRNA gene and the fungal ITS gene. To analyze bacterial diversity, with the use of universal primers 343F (5’-TACGGRAGGCAGCAG-3’) and 798R (5’-AGGGTATCTAATCCT-3’) (Lu et al. 2015), the V3-V4 variable regions of 16S rRNA genes were amplified. To analyze the diversity of fungi, using the universal primers ITS1F (5’-CTTGGTCATTTAGAGGAAGTAA-3’) and ITS2(5’-GCTGCGTTCTTCATCGATGC-3’) (Mukherjee et al. 2014), the ITS1 variable sections of ITS genes were amplified.

Library construction and sequencing: Using agarose gel electrophoresis, the Amplicon quality was examined. The PCR products were amplified for a second cycle of PCR after being purified using AMPure XP beads (Agencourt). The resulting amplicon was quantified using Thermo Fisher Scientific’s Qubit dsDNA Assay Kit after being once again purified with AMPure XP beads. A 250 bp paired-end Illumina NovaSeq 6000 was used for the sequencing process.

Raw sequence processing: FASTQ format was used for the raw sequencing data. Using Trimmomati (Bolger et al. 2014) software, paired-end readings were subsequently preprocessed to find and remove ambiguous bases. Using FLASH software, paired-end readings were put together after cutting (Reyon et al. 2012). By eliminating reads that comprised ambiguous or homologous sequences or that were less than 200 bp, the sequences were further denoised. Reads with a base percentage of 75% or more were kept. Reads with chimera were detected and removed. Clean reads were subjected to primer sequence removal and clustering to generate amplicon sequence variants (ASVs) using Vsearch (Rognes et al. 2016) software with a 97% similarity criterion. Using the QIIME package, the representative read for each ASV was determined. All representative bacterial reads were annotated and blasted against Silva database Version 138 using the RDP classifier (Wang et al. 2007). All representative fungal reads were annotated and blasted against the Unite database using BLAST (Ingrid Lobo 2008).

### Volatile flavor compound analysis

By using GCMS-QP2010 (Shimadzu, Japan), the VFCs were found. Some modifications were made according to the previous research methods (Wu et al. 2023b). The CTL samples were milled with liquid nitrogen and mixed evenly. A 0.5 g sample was placed in a 20 mL headspace bottle containing 8 mL saturated sodium chloride solution and 10 μL (1.03 μg/μL) phenethyl acetate internal standard solution. The headspace bottle was balanced at 65 °C for 20 min. Subsequently, the 50/30 µm DVB/CAR/PDMS fiber was inserted into the headspace bottle and used to extract VFCs at 65 °C for 35 min. The next step was to insert the fiber head into the injection port for 5 min of analysis, with a DB-5MS column (30 m × 0.25 mm id × 0.25 µm film thickness). Helium was flowing at a rate of 0.9 mL per minute, with an inlet temperature of 250 °C. Meanwhile, temperatures for the ion source and transfer lines remained at 230 °C and 280 °C, respectively. The following steps were temperature procedures: the column oven’s temperature was held at 60 °C for 1 min, then increased to 180 °C at a rate of 6 °C per minute, held for 2 min, and then elevated to 260 °C at a rate of 2 °C per minute, held for 2 min. The EI voltage for the electron impact (EI) ionization mode was 70 eV. For full-scan mode, a mass scan range of 35–450 *m/z* was employed. The mass spectrum information of the detected VFCs was qualitatively determined with the NIST14 standard mass spectrum database, and each chemical underwent a quantitative examination using the internal standard approach.

### Statistical analysis

The data were statistically analyzed by ANOVA using SPSS software (version 22.0), and Duncan’s test was used to assess the mean difference at the significance level of *p* < 0.05. QIIME software (version 1.9.1) was used for alpha and beta diversity analysis. R software (version 2.15.3) was used to plot dilution curves to evaluate sequencing depth. Linear discriminant analysis effect size (LEfSe) was used to assess for significant taxonomic differences between fungi and bacteria. Groups whose abundance differed significantly between categories were identified using the Kruskal–Wallis rank-sum test and then used linear discriminant analysis (LDA) to assess the influence of significantly different species. The graph package in R was used to create correlation networks between microorganisms, and the networks were then displayed using the Gephi software (version 0.9.7), which was based on Spearman’s correlation matrices (Bastian et al. 2009). Pie charts and stacked charts were plotted through the Origin software (version 2018). Visualization of VFCs by heat maps with TBtools software (version 2.019). Partial least squares discriminant analysis (PLS-DA) was adapted to investigate the VFCs through the SMICA software (version 14.1). The graph package in R was used to create correlation networks between microorganisms and VFCs, and the networks were then displayed using the Cytoscape software (version 3.7.1), which was based on Spearman’s correlation matrices. The statistical analysis used standardized data for all of the inputs.

### Accession numbers

The raw sequence data were submitted to the Sequence Read Archive of the National Center for Biotechnology Information database under BioProject PRJNA990350 (BioSample accession numbers: SAMN36265917–SAMN36265940) and BioProject PRJNA990363 (BioSample accession numbers: SAMN36267047–SAMN36267070).

## Results

### A review of the microbiological findings

#### Analysis of microbial community diversity in CTLs of diverse origins

**Fig. 1:**
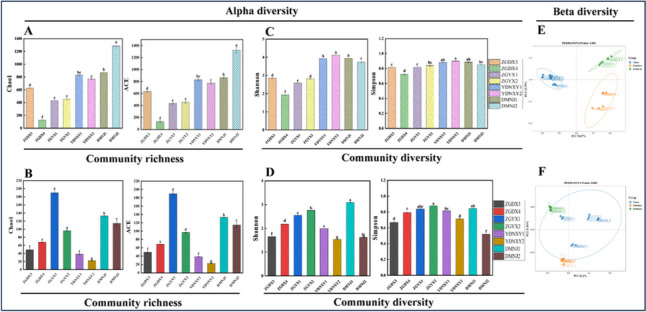
Microbial community diversity in CTLs from diverse origins. Bacteria alpha diversity (**A**, **C**) and fungal alpha diversity (**B**, **D**) of CTLs from diverse origins are determined based on the Chao1 index, the ACE index, the Shannon index, and the Simpson index. Considerable differences among various CTL samples are denoted by lowercase letters at the 0.05 level (*p* < 0.05). Bray–Curtis distance is employed to calculate the beta diversity of bacteria (**E**) and fungi (**F**)

Firstly, bacterial sequencing results revealed that eight samples yielded 2,007,994 high-quality sequences through three biological repetitions, ranging from 69,228 to 100,030 for each sample. Fungus sequencing revealed that 1,442,796 high-quality sequences, ranging from 42,179 to 72,161 for each sample, were obtained. All samples had a good coverage rate of more than 99.99%, which confirmed that the sequencing depth was sufficient to saturate microbial diversity, and most microorganisms had been captured, consistent with the sparse curve (Figure S1). The alpha diversity of CTLs from diverse origins was investigated using the Chao1, Ace, Shannon, and Simpson indices. The Chao1 and Ace indices were utilized to measure community diversity, the Shannon and Simpson indices were utilized to measure community richness. For bacterial alpha diversity, the richness and evenness of bacterial communities in Indonesian and Dominican CTLs were higher than those in China (Fig. 1A and C). DMNJ2 had the highest Chao1 and Ace indices of 1284.72 ± 62.65 and 1326.74 ± 92.29, respectively (Fig. 1A), and YDNXY2 had the highest Shannon and Simpson indices of 4.12 ± 0.08 and 0.9 ± 0.01, respectively (Fig. 1C). The diversity and abundance of fungal microorganisms also showed differences. Dominican CTLs still showed high microbial diversity and richness (Fig. 1B and D). DMNJ1 Shannon and Simpson indices were the highest with 3.09 ± 0.02 and 0.84 ± 0.00, respectively (Fig. 1D); ZGYX1 had the highest Chao1 and Ace indices of 190.00 ± 11.14 and 190.06 ± 11.23, respectively (Fig. 1B). These findings made it abundantly evident that the microbial communities of CTLs from various origins differ significantly from one another. The diversity and richness of bacterial microorganisms were higher than that of fungi.

To better understand the differences between the microbial communities of CTLs from different origins, beta diversity was explored by PCoA analysis based on the Bray–Curtis distance algorithm. PCoA analysis showed that the total bacterial and fungal interpretations were 82.86% and 84.01%, respectively, and Adonis analysis showed that the differences in the bacterial (*R*^2^ = 0.801; *p* = 0.001) and fungal (*R*^2^ = 0.597; *p* = 0.001) community compositions of CTLs from different origins both reached a significant level. The bacterial communities of different CTLs were clustered according to their origins, which were China, Dominica, and Indonesia, respectively (Fig. 1E). PCoA analysis of fungal community revealed that Dominican and Indonesian CTLs were clustered based on their origins, while Chinese CTLs were segregated on the horizontal axis, and the community varied greatly among the samples, with some similarity between ZHYX1 and ZHYX2 (Fig. 1F). As a whole, the microbial community composition of CTLs from different origins varied significantly, and the microbial community diversity of CTLs reflected the geographical differences of CTLs. The bacterial community was more obviously influenced by the origin.

#### Analysis of microbial community composition in CTLs from diverse origins

**Fig. 2:**
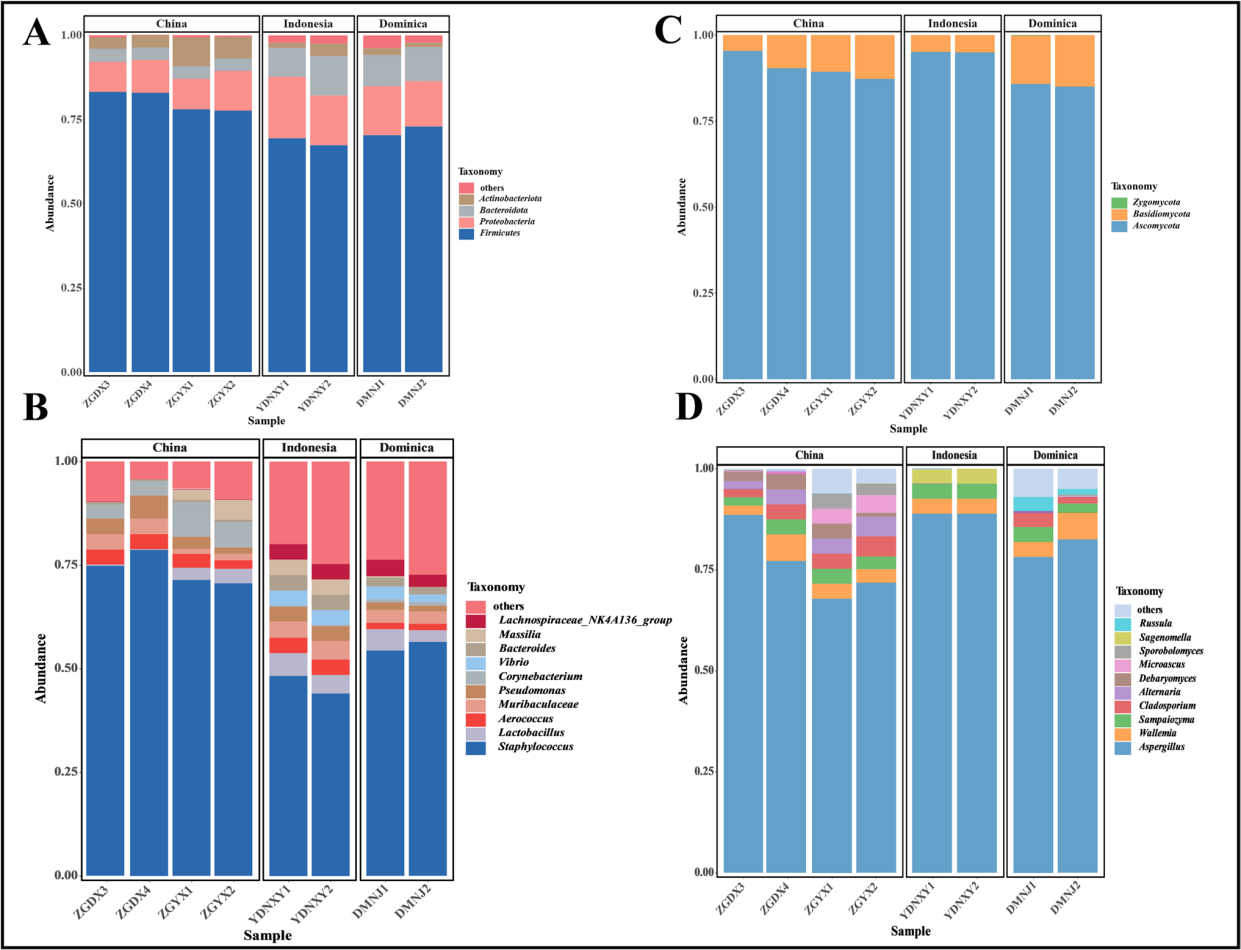
Microbial community composition in CTLs from diverse origins. Columnar stack diagrams at the phylum level for bacteria (**A**) and fungi (**C**). Columnar stack diagrams at the genus level for bacteria (**B**) and fungi (**D**)

The community composition of CTLs from various origins was identified at the phylum and genus levels by taxonomic analysis. In terms of the phylum of bacteria, 30 phyla were discovered via high-throughput sequencing of CTLs from different origins. The majority of the microorganisms belonged to four main phyla: *Firmicutes*, *Proteobacteria*, *Actinobacteria*, and *Bacteroidetes* (Fig. 2A). *Firmicutes* was dominant in all CTLs (relative abundance 69.46–86.77%). More than 99% of the bacterial microorganisms in Chinese CTLs mainly came from the above four phyla, while a certain number of bacterial microorganisms in CTLs from Indonesia and Dominica also came from *Spirochaetota*. In terms of the phylum of fungi, three phyla were detected: *Ascomycota*, *Basidiomycota*, and *Zygomycota* **(**Fig. 2C**)**. *Ascomycota* was dominant in all CTLs (relative abundance 85.00–95.39%).

Unlike the distribution of microorganisms at the phylum level, CTLs from different origins differ somewhat at the genus level. As depicted in Fig. 2B, in terms of the genus of bacteria, a total of 479 microorganisms were detected, of which 32 were more than 1% abundant. *Staphylococcus*, *Lactobacillus*, *Aerococcus*, *Muribaculaceae*, and *Pseudomonas* were the top 5 dominant bacterial genera in relative abundance; *Staphylococcus* was dominant in all eight samples (relative abundance 48.99–83.63%). *Corynebacterium* and *Pantoea* also were the dominant bacteria in CTLs from China (relative abundance > 1%); *Vibrio*, *Sphingomonas*, and *Bacteroides* were the dominant bacteria in Dominican and Indonesian CTLs. Dominican CTLs also contained a significant amount of *Bacillus*. In terms of the genus of fungi, a total of 135 microorganisms were detected, of which 15 were more than 1% abundant. *Aspergillus*, *Wallemia*, *Sampaiozyma*, *Cladosporium*, and *Alternaria* were the main genera **(**Fig. 2D**)**. *Aspergillus* was dominant in all CTLs (relative abundance 71.8% to 92.3%). The microbial community structure and relative abundance in CTLs can vary across different origins, bacterial communities are more richly composed compared to fungal communities.

#### Analysis of the difference of microbial species in CTLs from diverse origins

**Fig. 3:**
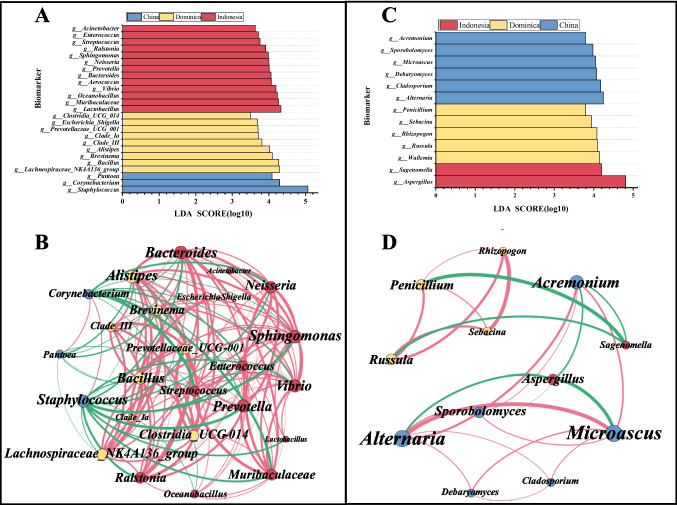
Difference of microbial species in CTLs from diverse origins. Bacteria (**A**) and fungi (**C**) LDA value distribution bar plot at the genus level. Color represents species with notable variations in abundance across several groups; the length of the bars on the graph depicts the size of the influence of various species (*p* < 0.01, LDA score > 3). Network analysis of bacterial (**B**) and fungal (**D**) communities in CTLs. The interaction with absolute correlation coefficient |*r*|> 0.7 and *p* < 0.05 is selected to draw the network analysis diagram, positive correlations are marked in red, and negative correlations are marked in green. The thickness of the line indicates the size of the interaction, and the thicker the line, the stronger the interaction

To conduct a more in-depth analysis of the variations in microbial communities in CTLs from diverse origins, biomarkers with statistical differences between different origins were identified based on sample community abundance data. The Kruskal–Wallis rank-sum test was utilized to identify microorganisms with significant abundance differences among different origins(*p* < 0.01); 150 bacterial genera and 18 fungal genera were found to be significantly different based on the findings (Figure S2). The impact of differential species was further assessed by linear discriminant analysis (LDA > 3.0), and statistically differentially high abundance biomarkers were identified. The results showed that bacterial biomarkers belonged to 5 phyla, 7 classes, 14 orders, 23 families, and 25 genera, and fungal biomarkers belonged to 2 phyla, 7 classes, 11 orders, 11 families, and 13 genera (Figure S3). The bacterial communities differed more than the fungal communities in CTLs of different origins, and it was these 38 microorganisms that drove the origin differentiation of CTLs. The magnitude of the LDA value represented the magnitude of the effect of the significantly different species on the differences between the groups, with the larger LDA value having a greater effect. Among them, the bacterial genus with the largest LDA value was *Staphylococcus* (Fig. 3A), and the fungal genus with the largest LDA value was *Aspergillus* (Fig. 3C), indicating that they had a strong influence on the origin differentiation of CTLs. In addition, Indonesian CTLs had the most specific bacteria, and Chinese CTLs had the most specific fungi.

Network analysis was further utilized to analyze the interactions between the above-found biomarkers. As shown in Fig. 3B, Spearman’s correlation analysis was performed on 25 bacteria, and a total of 147 relationships were found, of which 115 were positively correlated and 32 were negatively correlated. Based on the results of the above LEfSe analysis, the collinear network analysis was divided into three modules representing the significant differences in microorganisms enriched in CTLs of the three origins, with blue module indicating Chinese CTLs, yellow module indicating Dominican CTLs, and red module indicating Indonesian CTLs. *Corynebacterium*, *Pantoea*, and *Staphylococcus* belonged to the blue module; they showed cooperative relationships in the module and antagonistic relationships with other modules. *Staphylococcus* showed a significant negative correlation with most bacteria. Bacteria in the red module and the yellow module showed a significant positive correlation between their intra-group and inter-group relationships. Among them, *Vibrio*, *Sphingomonas*, *Bacillus*, *Alistipes*, *Bacteroides*, and other microorganisms had a strong synergistic relationship with most bacteria.

Similarly, Spearman’s correlation analysis was performed on 13 fungi, and a total of 24 relationships were found, of which 17 were positively correlated and 7 were negatively correlated (Fig. 3D). *Aspergillus* and *Sagenomella* belonged to the same module, but there was no obvious interaction between them, and they showed negative correlation with other modular fungi. The microorganisms of the same yellow module and the blue module showed a significant positive correlation inside the module, and there was no obvious relationship between the two modules. Microorganisms interacted with each other in a complex and predominantly positive way, affecting the entire microbial ecological network. Modularization analyses indicated closer relationships and stronger interactions within and between bacterial community modules.

### A review of the metabolomic findings

#### Characteristic analysis of VFCs in CTLs from diverse origins

**Fig. 4:**
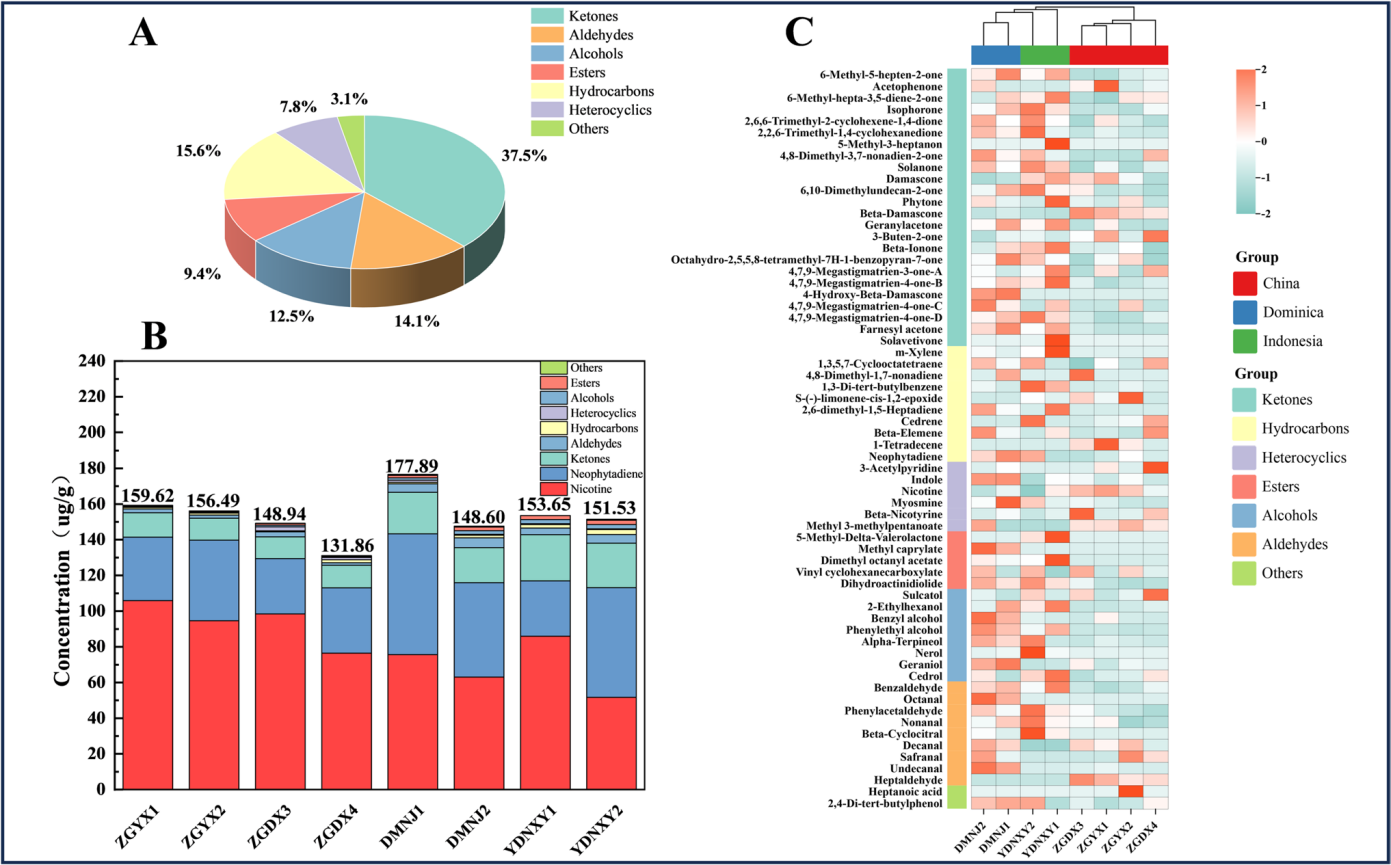
Composition analysis of VFCs in CTLs from diverse origins. **A** Pie chart of the proportion of VFCs in CTLs. **B** Accumulation map of VFCs types and contents in CTLs. **C** Cluster heat map of VFCs in CTLs, color represents the relative expression level of this metabolite in this group of samples; the matching relationship between color gradient and numerical size is displayed by the gradient color block

A total of 64 VFCs were detected in CTLs from diverse origins, and they were divided into 7 categories according to their structure, including 24 ketones (37.5%), 10 hydrocarbons (15.6%), 9 aldehydes (14.1%), 8 alcohols (12.5%), 6 esters (9.4%), 5 heterocyclics (7.8%), and 2 others (3.1%) (Fig. 4A). As displayed by the heat map (Fig. 4C), the types of VFCs in CTLs from diverse origins were similar, but the contents of each compound were different. The VFCs with the highest content were ketones (excluding hydrocarbons and heterocyclics where neophytadiene and nicotine were located), followed by aldehydes, alcohols, and esters. The contents of ketones and esters in Indonesian CTLs were the highest, and the contents of alcohols and aldehydes in Dominican CTLs were the highest (Fig. 4B).

A total of 24 ketones were detected in CTLs, among which 6-methyl-5-hepten-2-one, isophorone, solanone, 4,7,9-megastigmatrien-3-one, and beta-ionone were abundant; they exerted an enormous function on the formation of flavor characteristics of CTLs. It was worth noting that neophytadiene, as a significant characteristic VFC in CTLs, accounted for more than 20% of the total flavor quality of CTLs. The content of neophytadiene in Dominican CTLs was the highest, while the content of neophytadiene in Chinese CTLs was generally low. Nicotine was the most abundant heterocyclics in CTLs, and also the most abundant VFCs, accounting for more than 30% of the total content of VFCs in CTLs, and the content of nicotine from China CTLs was significantly higher than CTLs of other origins.

#### Analysis of the difference of VFCs in CTLs from diverse origins

**Fig. 5:**
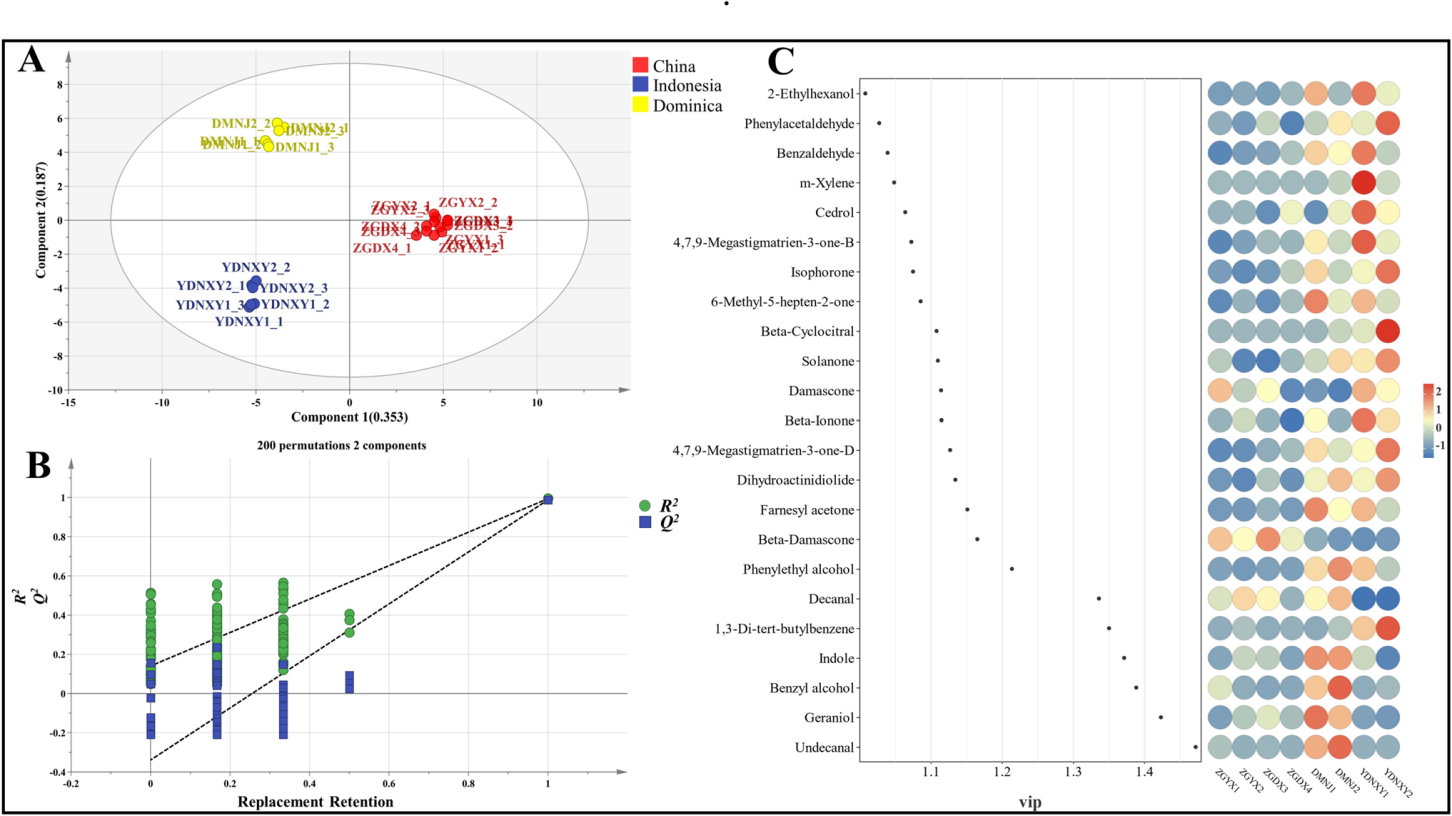
Analysis on the difference of VFCs in CTLs from diverse origins. **A** PLS-DA analysis of VFCs in CTLs from different origins, *R*_x_^2^ is 0.54, *R*_y_^2^ is 0.986, and *Q*^2^ is 0.97. **B** Permutation test of permutation test of PLS-DA model. **C** Point-and-stick heat map of 23 different VFCs (*p* < 0.05, VIP > 1.0). A VIP bubble map of the metabolites is displayed on the left, ordered from top to bottom by VIP value; the *X*-axis is the projection of the variable importance of each VFC based on the PLS-DA model (VIP > 1.0); the metabolite expression is shown on the right; the color denotes the metabolite’s relative expression level in this sample set. The link between color gradient and numerical size is demonstrated by the color gradient block

The VFCs of CTLs from diverse origins were analyzed differently by the PLS-DA model, and the CTLs were clustered according to their origins (Fig. 5A). The variable fitting index and the model prediction index exceeded 0.5, indicating that the model fitting results were acceptable. Figure 5B demonstrates that the model validation was effective and can be used for the origin identification analysis of CTL flavor. With the help of the VIP values of VFCs derived from the PLS-DA model to measure the degree of influence of each VFC on the discrimination of differences in CTLs, we further identified the VFCs that played a key role in the differentiation of the origin of CTLs and screened 23 different VFCs according to the criterion of *p* < 0.05 and VIP > 1.0. Among them, the most prominent VFCs in Indonesian CTLs were beta-ionone, damascone, solanone, benzaldehyde, and cedrol, while the most prominent VFCs in Dominican CTLs were indole, geraniol, benzyl alcohol, undecanal, and farnesyl acetone; the most prominent VFCs in Chinese CTLs were beta-damascone and damascone (Fig. 5C). Most of these VFCs had pleasant aromas and played a great role in harmonizing, modifying, and differentiating the overall aroma of CTLs from different origins.

Determination of CTLs flavor profile depends on VFCs with high OAV (odor activity value). OAV is the ratio of the mass concentration of VFCs to their sensory threshold (Hao et al. 2023). A VFC is deemed to have a discernible impact on the flavor characteristics of CTLs when its OAV exceeds 1, and a VFC is considered to significantly contribute to the overall flavor profile of CTLs when its OAV exceeds 10 (Shao et al. 2023). According to the threshold value and attribute description of VFCs reported in the literature, a comprehensive assessment of 23 VFCs was conducted to calculate their OAV in CTLs from various origins. As displayed in Table S1, among these VFCs, the OAV of 16 VFCs was greater than 1, indicating their potential significance in influencing the flavor attributes of CTLs sourced from diverse regions. The OAV of phenylacetaldehyde, cedrol, and damascone were higher than 10, and the OAV of beta-ionone, beta-damascone, 4,7,9-megastigmatrien-3-one-B were even higher than 100, suggesting that these VFCs may serve as pivotal components contributing to the distinctive flavor profile of CTLs.

The more prominent VFCs of OAV in Chinese CTLs were beta-damascone and decanal, which gave CTLs a strong floral and fruity flavor. The VFCs with high OAV in Dominican CTLs were undecanal, geraniol, indole, 6-methyl-5-hepten-2-one, and 4,7,9-megastigmatrien-3-one-B; they were mostly floral, fruity, and sweet. The VFCs with high OAV in Indonesian CTLs were mostly floral, fruity, and woody and accompanied by a slight bean aroma, including dihydroactinidiolide, beta-cyclocitral, beta-ionone, damascone, cedrol, and phenylacetaldehyde. CTLs of different origins had VFCs with different high OAV and therefore had different flavor characteristics.

#### Correlation analysis of microorganisms and VFCs in CTLs

**Fig. 6:**
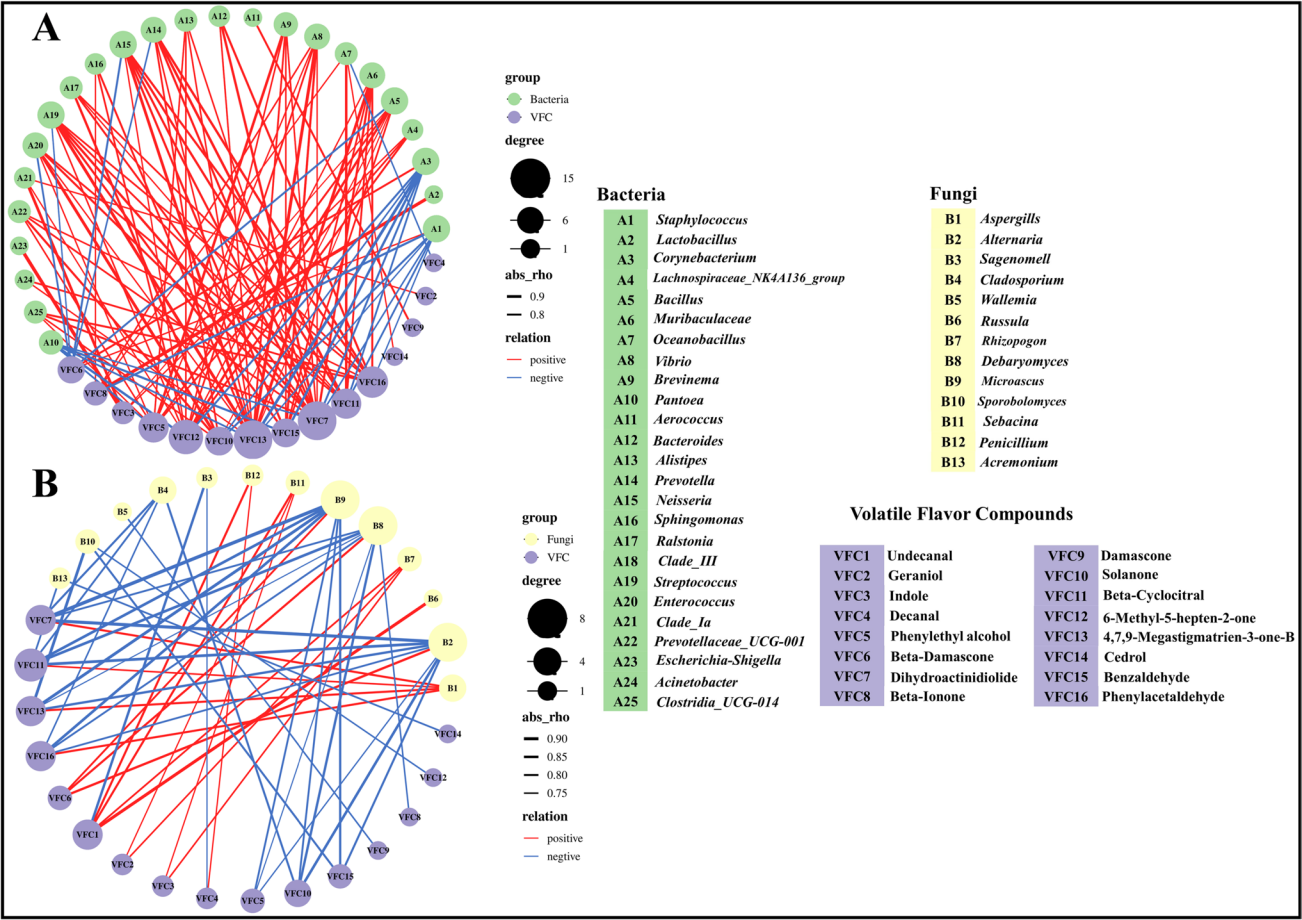
Correlation analysis of VFCs with bacterial (**A**) and fungal (**B**) communities. Through calculating the Spearman correlation coefficient, the interaction with absolute correlation coefficient |*r*|> 0.7 and *p* < 0.05 is selected to draw the network correlation graph; blue denotes a negative correlation, whereas red denotes a positive correlation. Circles represent microorganisms and VFCs; the larger the circle, the stronger the correlation between microorganisms and VFCs

The involvement of microorganisms is crucial in the development of flavor characteristics in CTLs. To enhance our understanding of the intricate relationship between microbial communities and VFCs in CTLs, selected microorganisms (25 bacteria and 13 fungi, Fig. 3A and C) identified by microbial community difference analysis were plotted against 16 VFCs in a correlation network diagram. In the correlation analysis of bacteria and VFCs, the vast majority of bacteria and VFCs showed significant positive correlation (Fig. 6A). *Staphylococcus* and *Corynebacterium* were positively correlated with beta-damascone and negatively correlated with other associated VFCs; *Pantoea* also showed negative correlation with all associated VFCs. All three bacteria were significantly enriched in Chinese CTLs, belonged to the same module in the microbial network analysis, and had a synergistic relationship (Fig. 3B). They acted synergistically to affect VFCs, which may explain the higher content of beta-damascone in Chinese CTLs. Twenty-one bacterial microorganisms, including *Bacillus*, *Vibrio*, *Sphingomonas,* and *Bacteroides*, were positively correlated with 14 VFCs. *Aerococcus* had a strong positive correlation only with damascone. *Oceanobacillus* was significantly positively correlated only with cedrol and significantly negatively correlated with decanal. Dihydroactinidiolide, 4,7,9-megastigmatrien-3-one-B, and 6-methyl-5-hepten-2-one were more closely related to microorganisms.

Whereas the relationship between fungal microorganisms and VFCs was more negative correlation (Fig. 6B). *Aspergillus*, *Russula*, *Rhizopogon*, *Sebacina*, and *Penicillium* were positively correlated with 8 VFCs; other fungi were negatively correlated with VFCs. *Alternaria*, *Microascus*, and *Debaryomyces* showed a negative correlation with phenylethyl alcohol, dihydroactinidiolide, solanone, beta-cyclocitral, 4,7,9-megastigmatrien-3-one-B, and phenylacetaldehyde, but a significant positive correlation with beta-damascone. Microbial communities were closely related to VFCs; multiple microorganisms worked together to influence VFCs. Bacteria were more closely related to VFCs and tended to be more positively correlated.

#### Analysis of carotenoid metabolic pathways of different VFCs

**Fig. 7:**
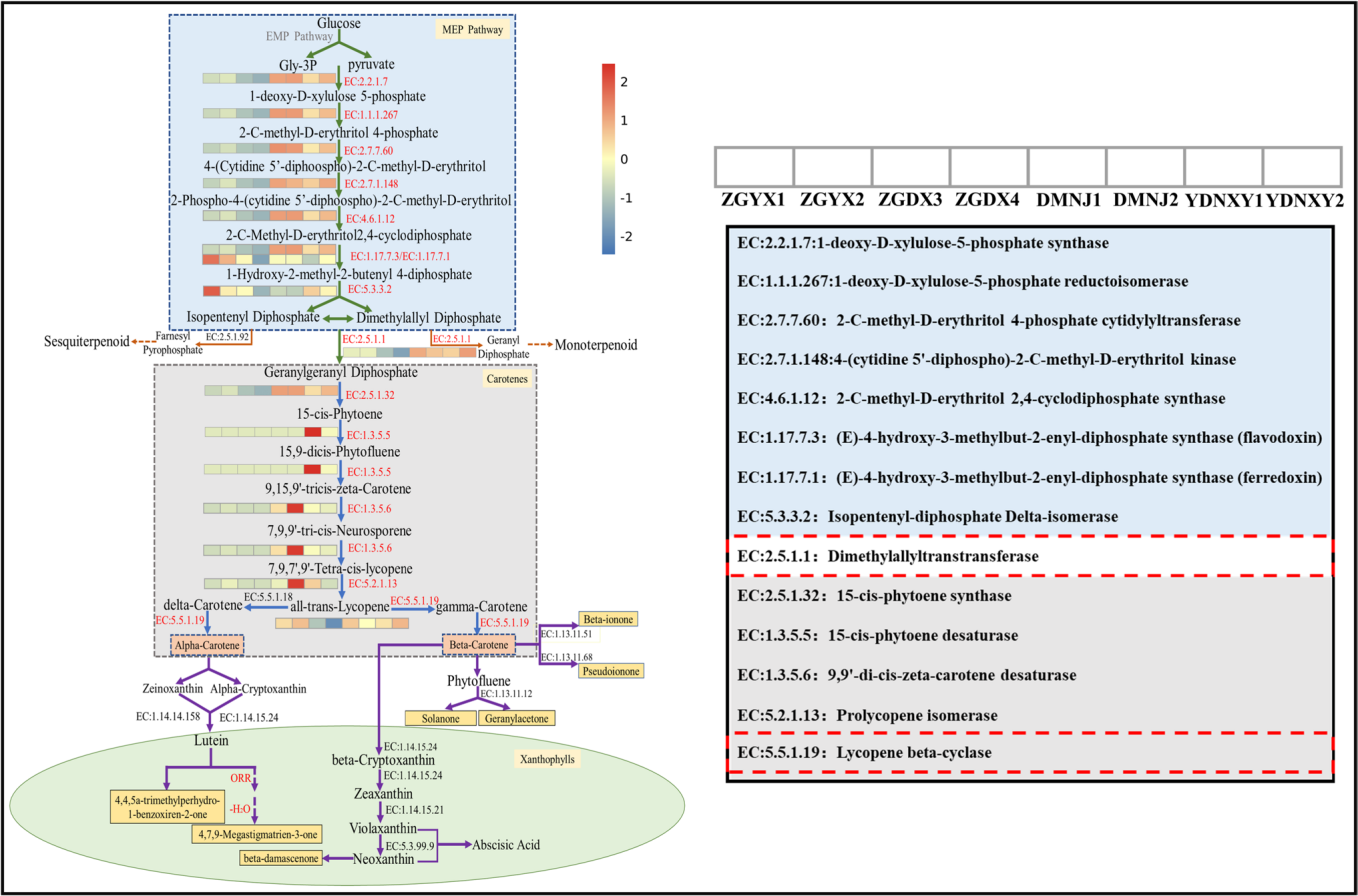
The key metabolic pathways involved in different VFCs in CTLs (the carotenoid biosynthesis pathway and its transcriptional control). The pathway in the blue rectangle above represents the production of substrates for carotenoid synthesis through the MEP pathway. The pathway in the gray rectangle and green ellipse represents carotenoids’ creation and breakdown in plastids. The abundance of enzymes is indicated by heat map

Through the analysis of key differential VFCs in CTLs from different origins, it was found that most of the key VFCs belonged to the degradation products of carotenoids. The carotenoid degradation pathways connected to flavor metabolites in CTLs from various origins were identified using KEGG analysis based on PICRUSt2. As shown in Fig. 7, the MEP, carotenes, lutein, and the key enzymes involved in them were listed. In the MEP pathway, glyceraldehyde triphosphate and pyruvate produced by glucose through glycolysis were the initiating substances of the MEP pathway. Next, the synthesis of carotenoid precursors was promoted by the action of various enzymes secreted by microorganisms. Among them, the enzyme abundance of Dominican CTLs was higher, indicating that the synthesis of precursors of carotenoids involved in Dominican CTL microorganisms was more intense. Geranylgeranyl pyrophosphate synthase (EC 2.5.1.1) was a key enzyme that catalyzed the conversion of isopentenyl diphosphate (IPP) and dimethylallyl diphosphate (DMAPP) into geranylgeranyl diphosphate (GGPP) for carotenoid synthesis. Dominican and Indonesian CTLs had higher EC 2.5.1.1 abundance, which facilitated GGPP synthesis. In addition to being common substrates for carotenoid synthesis, IPP and DMAPP can also be transformed into monoterpenoids and sesquiterpenoids. Therefore, controlling the flow direction of IPP and DMAPP was an important means of aroma improvement (Rasouli et al. 2018).

In the carotenoid synthesis pathway, lycopene cyclization was a crucial branch point in the biosynthetic process for carotenoids. Lycopene beta-cyclase (EC 5.5.1.19) is the key enzyme that facilitates the production of beta-carotene and alpha-carotene from lycopene, which can further produce a variety of aromatic ketones, such as beta-ionone, solanone, and geranylacetone. Indonesian CTLs had a higher abundance of beta-cyclase; the metabolic activity involved in the production of alpha-carotene and beta-carotene was more active. Alpha-carotene and beta-carotene were important precursors of lutein synthesis; lutein can also be further transformed into important aroma substances such as 4,7,9-megastigmatrien-3-one and beta-damascone. According to the above analysis, substance metabolism in the MEP pathway and carotene synthesis pathway in Dominica CTLs was more active, providing abundant precursor substances for the synthesis of VFCs, and significantly increasing the content of aromatic substances of carotenoid degradation products.

## Discussion

Tobacco, as a widely grown non-food crop in the world, has distinctive regional characteristics in terms of flavor quality due to differences in environmental conditions in different regions. Moreover, it is also crucial to recognize that the fermentation process and the impact of microorganisms play a pivotal role in shaping the flavor and overall quality of CTLs. These factors significantly contribute to the sensory profiles and market value of tobacco products. Hence, this paper focuses on microorganisms and flavors.

By analyzing the microbial communities, it can be seen that the diversity and richness of bacterial community were greater than that of fungi according to the Alpha diversity indices (Fig. 1), and the microbial communities of CTLs from different origins differed significantly at the genus level (Fig. 2). The dominant bacteria in CTLs of different origins mainly included *Staphylococcus*, *Lactobacillus*, *Aerococcus*, *Pseudomonas*, and *Muribaculaceae* (Fig. 2B). When CTLs are fermented in piles, *Aerococcus* and *Staphylococcus* can utilize the sugars and acids in the leaves, leading to an increase in the temperature and pH of the piles, which in turn promotes the growth of salt-tolerant and resilient *Corynebacterium* (Di Giacomo et al. 2007), thus driving changes in the structure of the microbial community, and *Corynebacterium* was also the dominant microorganism in the present study. *Staphylococcus* is also related to the production of protease and lipase, which can promote the production of VFCs (Hu et al. 2019; Wang et al. 2022). Likewise, *Lactobacillus* can also digest and metabolize proteins and carbohydrates, producing a range of functional compounds that improve the flavor (Bancalari et al. 2020). Some species of *Pseudomonas* have strong protein degradation ability (Mariencheck et al. 2003) and can also degrade nicotine, affecting the smoking vigor of CTLs (Ma 2012). The dominant fungi in CTLs were mainly *Aspergillus*, *Wallemia*, *Sampaiozyma*, *Cladosporium*, and *Alternaria*. *Aspergillus* occupied a dominant position in the entire microbial community. *Aspergillus* can promote the degradation of sugars and proteins and the synthesis of flavor compounds in tobacco (Mu et al. 2020). *Sampaiozyma* has been shown to degrade carotenoids and therefore shows potential value in flavor (Huang et al. 2022). *Cladosporium* and *Alternaria* are common saprophytic fungi in tobacco, which can easily cause mold and damage the quality of tobacco. The dominant microorganisms are capable of decomposing, transforming, metabolizing, and utilizing the matrix components of CTLs, thereby playing a crucial role in determining their quality characteristics (Costa et al. 2020).

The microbial communities of CTLs sourced from diverse origins exhibited considerable dissimilarity at the genus level, encompassing variations not only in the relative abundance of dominant microorganisms but also in the overall community structure. Geographic differences in the microbial community of CTLs were revealed by PcoA analysis (Fig. 1E and F), and LEfSe analysis also showed that the presence of highly abundant differential microbes drove the clustering of origins of CTLs (Fig. 3A and C). CTLs from different origins had different highly abundant differential microorganisms. The environmental microbial community is the main source of the microbial composition of CTLs, so differences in geospatial location result in differences in the tobacco microbial community (Kandel et al. 2017). In addition, the change of process parameters will also drive the succession of microbial communities and cause the dynamic fluctuation of microbial communities when CTLs are processed by fermentation (Han et al. 2020; Liang et al. 2019). Through complex interactions, microorganisms from different origins worked together to influence the structure and function of the entire community, which in turn influenced the flavor quality of CTLs.

The formation of flavor characteristics in CTLs was influenced by the types and contents of VFCs. Most of the detected VFCs belonged to ketones, hydrocarbons, aldehydes, and esters, of which ketones were the most important **(**Fig. 4A**)**. Ketones have an aromatic carbonyl group in their chemical structure, and most compounds with carbonyl groups have a lovely scent (Shen et al. 2019). Such as the detected VFCs, beta-ionone, beta-damascone, and 6-methyl-5-hepten-2-one gave CTLs fruity and floral characteristics. Some heterocyclics, such as 3-acetylpyridine and Indole, gave CTLs a nut aroma and baking aroma. Aldehydes gave CTLs a woody, slightly oily aroma. Esters can bring sweet and fruity flavors to CTLs. CTLs from different origins contain different types of prominent VFCs, and therefore their flavor profiles vary somewhat (Fig. 4B).

It is worth noting that CTLs of different origins are the richest in both neophytadiene and nicotine. Neophytadiene is a degradation product of chlorophyll, which can carry aroma substances into the smoke and is an important flavor enhancer for CTLs (Hu et al. 2023). It also undergoes thermal cracking when burned at high temperatures, producing a large number of hydrocarbons, alcohols, ketones, aldehydes, and esters (Hu et al. 2022). The higher content of neophytadiene in Dominican CTLs may provide a more favorable impact on its flavor profile. Nicotine is related to the smoking flavor of tobacco; the right amount of nicotine will make the tobacco smoking taste full and pleasurable to the body, but too much nicotine will make the smoking irritation increase(Patten and De Biasi 2020). Chinese CTLs may face the problem of increased taste irritation due to high nicotine content. High OAV VFCs gave CTLs a more pronounced flavor profile; cedrol, phenylacetaldehyde, 4,7,9-megastigmatrien-4-one-B, damascone, beta-damascone, and beta-ionone had particularly high OAV in CTLs of different origins, and they were prominent contributors to the flavor of CTLs (Table S1). Dominican CTLs contained more VFCs with high OAV, giving them a more diverse and prominent flavor profile.

It is interesting to note that the 16 VFCs with OAV > 1.0 mentioned above were mostly carotenoid degradation products. Degradation of carotenoids by enzymes or under light and heat conditions produces several aromatic substances such as beta-damascone, damascone, and beta-ionone, which have a significant impact on the flavor quality and color of tobacco (Meléndez-Martínez et al. 2023). According to the analysis of the metabolic pathway of carotene in CTLs from diverse origins (Fig. 7), it has been observed that variations in the microbial community structures also influence the metabolic processes within CTLs; bacteria and fungi work together to produce a high abundance of enzymes for material transformation (Zhang et al. 2023b). The MEP pathway and the carotenoid synthesis pathway played important roles in the formation of flavor in CTLs. The MEP pathway is primarily responsible for the synthesis of carotenoids; geranylgeranyl pyrophosphate synthase (EC 2.5.1.1) is the key enzyme in this phase. It has been shown that by rationally designing geranylgeranyl pyrophosphate synthase, the carotenoid and chlorophyll contents in tobacco can be increased, enhancing its photosynthetic efficiency and improving its ability to withstand intense light adversity stress (Dong et al. 2022). In the carotenoid synthesis pathway, lycopene beta-cyclase (EC5.5.1.19) is the key enzyme driving the production of aromatic ketones. In addition to carotenoid degradation products, phenylethyl alcohol, benzaldehyde, and phenylacetaldehyde are phenylalanine degradation products, and in-depth analysis of the phenylalanine degradation pathway is also of great significance to the study of tobacco flavor formation.

By conducting a network correlation analysis of microorganisms and VFCs (Fig. 6), a stronger and predominantly positive correlation was found to exist between bacterial communities and VFCs. *Bacillus*, *Vibrio*, *Sphingomonas*, *Bacteroides*, and other microorganisms were closely related to VFCs and made significant contributions to the formation of CTL flavor. Studies have shown that *Bacillus* can produce glycosidase to promote the degradation of carotenoids in CTLs and contribute to the formation of CTLs aroma (Haure et al. 2022; Wu et al. 2021), *Sphingomonas* can break down polyphenols in CTLs, and the intermediate product of its metabolism of chlorogenic acid is aromatic organic acid, which is an important aroma precursor (Feng et al. 2018). The relationship between fungi and VFCs exhibited a stronger negative correlation. *Alternaria*, *Cladosporium*, and *Acremonium* were commonly found as saprophytic fungi in CTLs, which can easily lead to mold formation and hurt the flavor of CTLs. In addition, the combination of microbial network analysis showed that microorganisms often worked together to influence the flavor of tobacco. Therefore, when searching for functional microorganisms to enhance the flavor quality of CTLs, it is important to consider their synergistic effects on CTL quality (Domínguez-Tornay et al. 2024).

In conclusion, due to geographical differences and different fermentation processes, the microbial communities and VFCs of CTLs from different origins varied greatly. The bacterial community in CTLs was more abundant and diverse than fungi. Participation of dominant microorganisms in complex biochemical reactions plays an important role in the formation of characteristic qualities of CTLs. Sixteen VFCs with OAV greater than 1 were screened from CTLs of different origins, differences in high OAV VFCs in CTLs from different origins contribute to the geographic diversity of CTL flavors. The MEP pathway and the carotenoid synthesis pathway were key metabolic pathways for the formation of VFCs in CTLs. The interactions between microorganisms jointly contributed to the formation of CTL flavor, and the bacterial community was more closely linked to key VFCs, with bacteria such as *Bacillus*, *Vibrio*, and *Sphingomonas* contributing significantly to CTL flavor formation. CTL flavor formation is also related to the combined effects of amino acids, sugars, and nitrogen compounds, which can be further analyzed by liquid chromatography tandem mass spectrometry (LC–MS) technology and combined with sensory quality evaluation. Based on this study, the high-quality microbial resources in CTLs are further explored to be applied in obtaining tobacco flavor compounds. New CTL fermentation is developed to enhance the flavor quality of CTLs and promote the utilization of raw materials of CTLs.

## Supplementary Information

Below is the link to the electronic supplementary material.

Supplementary file1 (PDF 945 KB)

## Data Availability

The publication contains all the supporting information for the study’s findings, and the corresponding author is also willing to provide it upon reasonable request.
