# Supplementary material for: Correlation study on microbial communities and volatile flavor compounds in cigar tobacco leaves of diverse origins
Source: Appl Microbiol Biotechnol. 2024 Feb 26;108(1):236. doi: 10.1007/s00253-024-13032-6 (PMC10896874; doi:10.1007/s00253-024-13032-6)
Supplement: Supplementary file 1 — Supplementary file1 (PDF 945 KB) [file 253_2024_13032_MOESM1_ESM.pdf]

Correlation Study on Microbial Communities and Volatile Flavor Compounds in Cigar Tobacco Leaves of Diverse Origins

Haiqing Wang[1#], Dongfeng Guo[2*#], Mingzhu Zhang[1], Guanglong Wu[1], Yaqi Shi[2], Jinglong Zhou[2], Naihong Ding[2], Xiangsong Chen[3], and Xingjiang Li[1*]

[1] Anhui Fermented Food Engineering Research Center, School of Food and Biological Engineering, Hefei University of Technology, Hefei City 230009, Anhui Province, P.R. China.

[2] China Tobacco Anhui Industrial Co., Ltd., Hefei City, 230009, Anhui Province, P.R. China.

[3] Institute of Plasma Physics, Hefei Institutes of Physical Science, Chinese Academy of Sciences, Hefei City 230009, Anhui Province, P.R. China.

[#] These authors contributed equally to this work.

* Corresponding author at:

Xingjiang Li, Anhui Fermented Food Engineering Research Center, School of Food and Biological Engineering, Hefei University of Technology, Danxia Road 485#, Zip Code:230601, Hefei City, Anhui

Province. China. E-mail: lixingjiang@hfut.edu.cn. Tel: 18919653872

Dongfeng Guo, China Tobacco Anhui Industrial Co., Ltd., Huangshan Road 606#, Zip Code 230088, Hefe City, Anhui Province, China. E-mail: gdf0221@163.com.

Journal name：

Applied Microbiology and Biotechnology

Supplement Figure

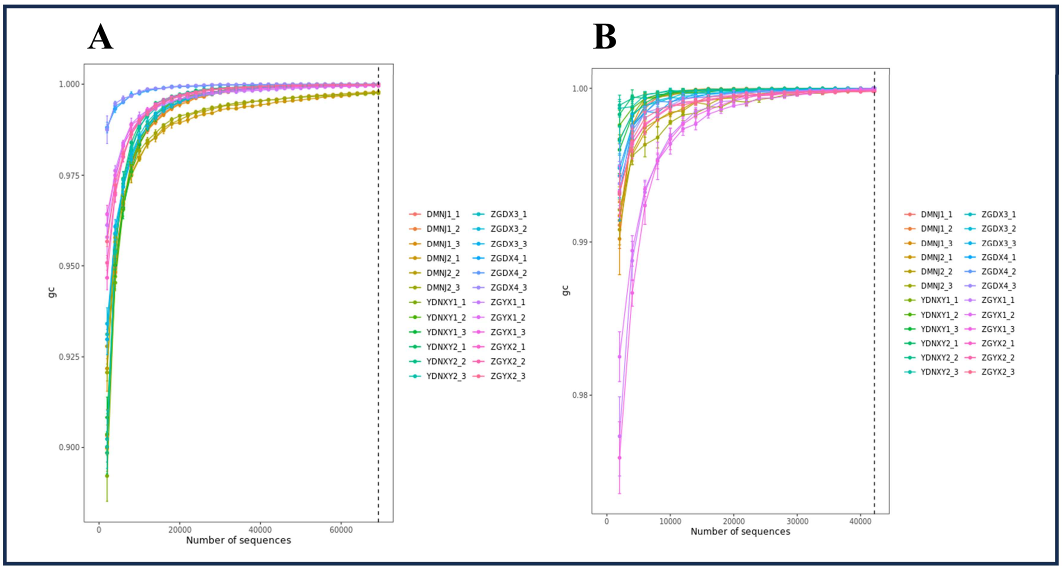

Fig. S1 Microbial communities diversity in CTLs from diverse origins. Bacterial (A) and fungal (B) sequencing deep dilution curves.

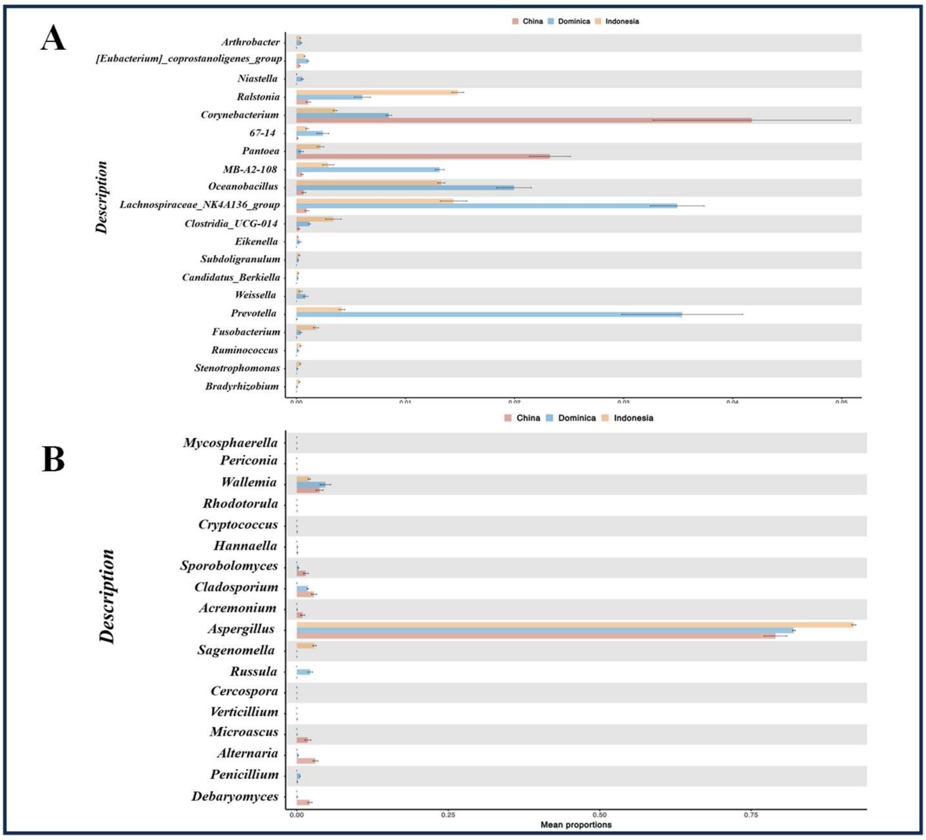

Fig. S2 Identification of bacteria (A) and fungi (B) with significant differences in the abundance of CTLs from different origins using the Kruskal-Wallis rank sum test, and the top 20 features selected for STAMP analysis are indicated(p < 0.01).

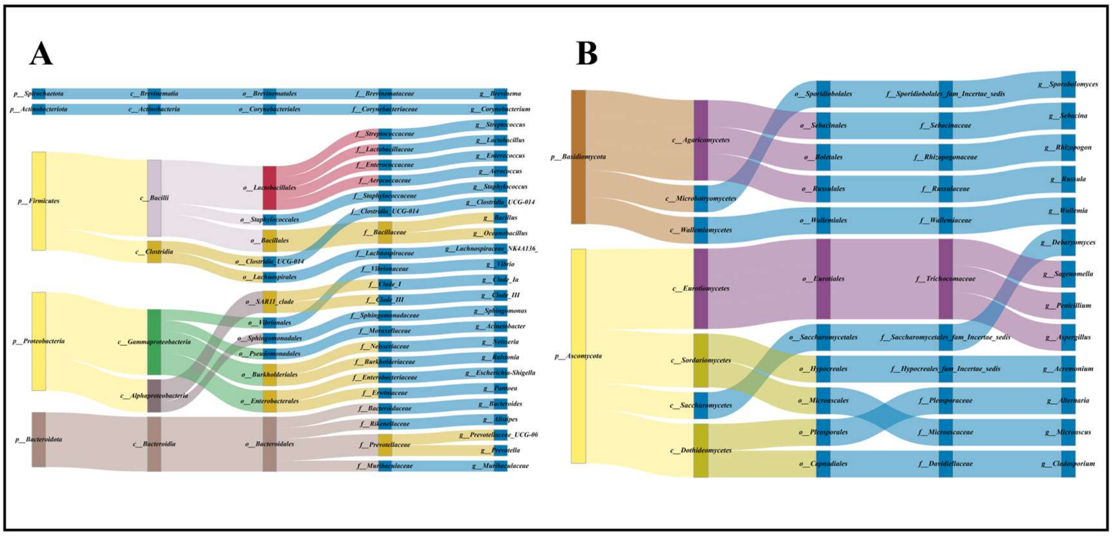

Fig.S3 Sankey diagrams of differential bacteria (A) and fungi (B) found by Lefse analysis at phylum, class, order, family, and genus levels.

Table S1 OAV of different VFCs in CTLs from diverse origins

Volatile Flavor Compound	Threshold (μg/g)	OAV(μg/g)								Description
		ZGYX1	ZGYX2	ZGDX3	ZGDX4	DMNJ1	DMNJ2	YDNXY1	YDNXY2	
Undecanal	0.0125	5.89±0.74^{c}	nd	1.82±0.74^{c}	nd	41.76±5.61^{b}	55.5±5.94^{a}	nd	nd	Sweet orange rose flowers
Geraniol	0.0066	11.76±1.05^{e}	27.36±4.59^{c}	39.19±11.89^{cd}	23.4±7.75^{de}	84.36±13.21^{a}	66.29±6.16^{b}	12.5±1.79^{de}	9.08±0.73^{e}	Sweet and rose
Indole	0.011	1.97±0.82^{c}	3.6±0.88^{b}	3.33±0.54^{b}	1.91±0.46^{c}	7.56±0.37^{a}	7.37±0.54^{a}	3.7±0.23^{b}	0.88±0.22^{c}	Flower
Decanal	0.0026	200.08±35.29^{c}	296.45±21.56^{a}	260.63±28.35^{b}	110.1±35.35^{d}	256.31±20.31^{b}	331±43.26^{ab}	nd	nd	Fruity, oily smell
Phenylethyl alcohol	0.479	0.15±0.02^{d}	0.06±0.03^{d}	0.15±0.02^{d}	0.13±0.01^{d}	1.36±0.16^{b}	1.87±0.08^{a}	1.51±0.07^{b}	0.57±0.1^{c}	Sweet rose flowers
Beta-Damascone	1.3×10^{-6}	864715.1±88630.52^{b}	654501.32±86335.81^{c}	1064643.41±98093.84^{a}	570539.41±46058.39^{c}	186342.68±17766.88^{d}	64993.02±8256.85^{e}	25999.29±5348.4^{e}	67425.52±8416.26^{e}	Rich rose aroma
Dihydroactinidiolide	0.28	1.17±0.39^{e}	0.63±0.16^{e}	2.85±0.28^{d}	0.96±0.18^{e}	4.55±0.43^{c}	6.48±0.38^{b}	4.66±0.28^{c}	7.42±0.46^{a}	Coumarin-like, musky
Beta-Ionone	0.007	115.7±8.81^{d}	170.15±6.37^{c}	114.04±5.67^{d}	33.33±9.14^{e}	220.97±11.88^{b}	111.2±7.89^{d}	349.3±49.92^{a}	251.21±9.79^{b}	Violet, wood
Damascone	0.007	96.29±15.76^{a}	57.02±11.01^{bc}	78.16±15.06^{ab}	27.07±8.71^{d}	33.49±15.15^{cd}	23.66±1.64^{d}	101.43±13.28^{a}	79.86±10.76^{ab}	Strong rose, fruity
Solanone	1.82	2.8±0.12^{cd}	2.04±0.09^{e}	1.95±0.05^{e}	2.6±0.08^{d}	2.96±0.19^{c}	3.67±0.15^{b}	3.55±0.1^{b}	4.22±0.21^{a}	Carrot, tobacco
Beta-Cyclocitral	0.003	nd	nd	nd	nd	nd	11.54±0.86^{b}	18.12±2.22^{b}	76.47±11.3^{a}	Fragrance, grass
6-Methyl-5-hepten-2-one	0.068	4.88±1.21^{e}	10.10±0.72^{d}	5.39±0.35^{e}	11.66±0.42^{d}	31.58±1.90^{a}	18.11±2.01^{c}	26.94±1.80^{b}	16.36±1.02^{c}	Fruity, fragrance
Cedrol	0.0005	266.75±51.72^{c}	278.85±43.34^{c}	35.45±5.99^{c}	665.5±165.15^{b}	48.39±11.65^{c}	649.26±208.25^{b}	1364.10±214.25^{a}	766.89±69.58^{b}	Woody sweetness

Volatile Flavor Compound	Threshold （μg/g）	OAV(μg/g)								Description
		ZGYX1	ZGYX2	ZGDX3	ZGDX4	DMNJ1	DMNJ2	YDNXY1	YDNXY2	
Benzaldehyde	0.75	0.41±0.11^{f}	0.63±0.13^{ef}	0.7±0.04^{e}	1.02±0.11^{d}	1.93±0.16^{b}	1.65±0.16^{c}	2.49±0.06^{a}	1.15±0.13^{d}	Bitter almond, cherry, nut
4,7,9-Megastigmatrien-3-one-B	0.0021	152.93±46.8^{g}	318.12±50.95^{f}	494.99±44.32^{e}	450.96±52.52^{e}	1020.68±33.94^{b}	685.96±39.84^{d}	1646.97±125.05^{a}	832.12±59.01^{c}	Sweet aroma
Phenylacetaldehyde	0.0063	81.45±16.77^{e}	49.4±13.54^{e}	139.54±12.95^{d}	14.33±0.7^{f}	131.92±13.34^{d}	228.63±14.4^{b}	178.72±17.58^{c}	367.93±29.89^{a}	Fruity sweetness, floral

"–" represents the odor was unknown, "nd" represents the substance is not detected, the same column of letters indicated the difference between VFCs in different samples (one-way analysis of variance; $p < 0.05$). The thresholds for VFCs are detected in water media, with reference to (Gemert 2003). The flavor description of VFCs are obtained from (Mao et al. 2020; Shi et al. 2023; Yu et al. 2021).

Reference

Gemert LJV (2003) Compilations of odour threshold values in air, water and other media.

Mao Z, Hong Z, Liu J, Zhou P, Yi Y, Cao X, Zhang X, Xie J (2020) Characteristic aroma components in tobacco extracts from Youxi based on odor activity value. Tobacco Science & Technology 53:56-65 doi:https://doi.org/10.16135/j.issn1002-0861.2020.0233

Shi Y, Pan Y, Du F, Zhao Z, Li Z, Wang Y, Yuan X, Zhang Z, Pang X, Wang J (2023) Identification and discrimination of characteristic aroma components of different cigar leaves based on static headspace/gas chromatog-raphy-ion mobility spectrometry combined with relative odor activity value and multivariate statistical analysis. Journal of Instrumental Analysis 42:674-683 doi:https://doi.org/10.19969/j.fxcsxb.22113001

Yu H, Liu Y, Shang M, Huang G, Fang Y, Lin L, Qu Y, Zuo Q (2021) Cigar leaf differences from different producing areas based on aroma component analysis. Tobacco Science & Technology 54:58-71 doi:https://doi.org/10.16135/j.issn1002-0861.2021.0003
